# The Incredible Adventure of Omalizumab

**DOI:** 10.3390/ijms25053056

**Published:** 2024-03-06

**Authors:** Christian Domingo, Daniel R. Monserrate, Ana Sogo, Rosa M. Mirapeix

**Affiliations:** 1 Department of Pulmonary Medicine, Parc Taulí Hospital Universitari, Institut d’Investigació i Innovació Parc Taulí (I3PT-CERCA), Universitat Autònoma de Barcelona, 08202 Sabadell, Spain; drmonserrate@tauli.cat (D.R.M.); asogo@tauli.cat (A.S.); 2 Unitat d’Anatomia, Departament de Ciències Morfològiques, Universitat Autònoma de Barcelona (UAB), 08193 Cerdanyola, Spain; rosa.mirapeix@uab.cat

**Keywords:** IgE, omalizumab, pivotal studies, real-life studies, safety, immunity, remodeling

## Abstract

The basis of our current understanding of allergies begins with the discovery of IgE in the mid-1960s. The whole theory of the physiology and pathophysiology of allergic diseases, including rhinitis and asthma, dates from that period. Among the key regions of IgE identified were the FAB (fragment antigen binding) portion that has the ability to capture allergens, and the Cε3 domain, through which IgE binds to its membrane receptor. It was then postulated that blocking IgE at the level of the Cε3 domain would prevent it from binding to its receptor and thus set in motion the allergic cascade. This was the beginning of the development of omalizumab, a monoclonal antibody with an anti-IgE effect. In this article, we review the pathophysiology of allergic disease and trace the clinical development of omalizumab. We also review the benefits of omalizumab treatment that are apparently unrelated to allergies, such as its effect on immunity and bronchial remodeling.

## 1. Introduction

In the early 20th century, asthma was classified as either extrinsic and intrinsic. The extrinsic form was identified with allergic asthma. In 1967, a new protein, immunoglobulin E (IgE), was discovered independently by Kimishige and Teruko Ishizaka in Denver, Colorado and by Bennich and Johansson in Uppsala, Sweden, and it was shown to cause allergic processes. The discovery of IgE, and later of specific IgE (IgE developed by the individual against a specific allergen), paved the way for the development of allergy vaccines during the last 25 years of the 20th century. Knowledge of the pathophysiology of asthma (especially early allergic asthma) expanded, disease markers were defined, and asthma phenotypes were described. All of this was linked to the development of new drugs known as biologics (so called because they were originally obtained from live Chinese hamster cells) or monoclonal antibodies (mAbs) [1]. Thanks to the knowledge obtained from the administration of immunotherapy for allergy, IgE took on special importance at the beginning of this process [2]. It was seen that the allergic process began with the activation of dendritic cells or antigen-presenting cells (APCs). The presence of allergens, antigens that favour the production and release of IgE to the medium by plasma cells, promotes the activation of the Th2 pathway, a pathway that requires the differentiation of Th0 lymphocytes (naïve T lymphocytes) into Th2 lymphocytes, and which is able to generate IgE against each allergen to which it is sensitized. This is why this process was called adaptive immunity (i.e., the production of the type of antibody “adapts” to the different allergens with which the individual has been in contact) [3]. As the cell initiating the reaction was the Th2 lymphocyte, the pathway was also called the Th2 pathway. The first monoclonal antibody to be marketed for bronchial asthma was omalizumab, a free IgE blocker [3,4]. Allergen-sensitized IgE-producing patients with clinical symptoms as a consequence of allergen exposure were termed the allergic phenotype.

Subsequently, it was learned that during this Th2 process other molecules known as interleukins (IL5, IL4, IL13) are released into the environment [5]. It has been confirmed that the presence of these interleukins favours, respectively, the synthesis, maturation, and recruitment of eosinophils, the activation of the Th2 pathway, and the blocking of the expression of vascular cell adhesion molecules (VCAM-1s), and finally the activation of the enzyme that regulates the release of the exhaled fraction of nitric oxide (FeNO), nitric oxide synthase [5].

More recently, it has been observed that apart from this adaptive pathway, the activation of the innate immunity also occurs, which is produced by other molecules: thymic stromal lymphopoietin (TSLP), IL33, and IL25 [1,5]. It was observed that these mediators appear as a result of damage to the bronchial ciliated epithelium, which releases them into the environment [1]. Since they represent an alarm signal warning of epithelial damage, they were named alarmins. This pathway is activated by the effects of different antigens (including allergens which would act in this case by inflammatory and non-allergenic means), and by pollution, tobacco, viruses and bacteria, etc., which damage the bronchial epithelium. This pathway always responds in the same way, thus differentiating it from the adaptive pathway in which the production of specific IgE against each allergen to which the individual is sensitized occurs. Because they are mediated by the innate lymphoid cells type 2 (ILC2), and as an analogy with the allergic cascade which was called Th2 due to the participation of Th2 lymphocytes, the innate and adaptive pathway as a whole were called T2; hence the classification of asthma into T2 and non-T2 [5].

These alarmins directly activate the cells of innate immunity, the ILC2 and the natural killer T cells (NKT cells). These alarmins, especially TSLP, exert control over the activity of antigen-presenting cells. T2 asthma (and perhaps non-T2 asthma) then becomes an epithelium-driven disease [1]. As mentioned above, anti-alarmin monoclonal antibodies are currently being developed [6]. When we administer a mAb, we are modifying the phenotype of the individual, and this causes the organism to try to maintain the lost balance by activating other pathways. We will now describe the scientific and clinical development of the first monoclonal antibody that was marketed two decades ago.

## 2. Structure of IgE

**Figure 1:**
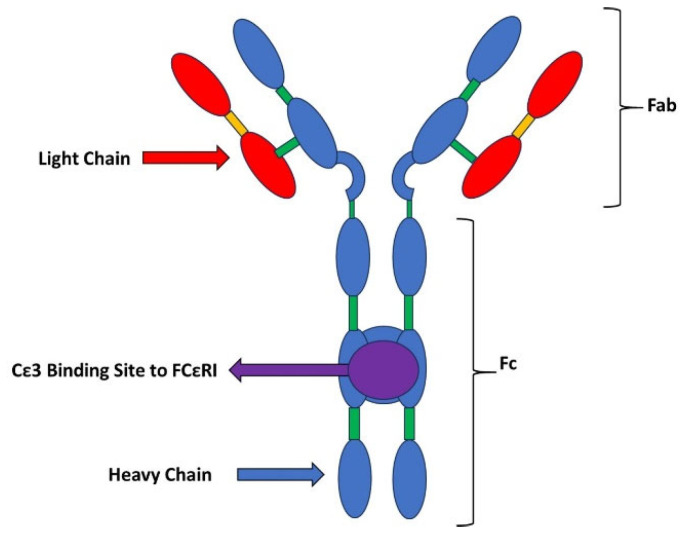
Structure of IgE (modified from [2]). The Cε3 domain is the fraction of the IgE molecule that binds to membrane receptors. The key epitope is the Cε3 domain of human IgE.

IgE has the structure shown in Figure 1. It has two light chains and two heavy chains. Of special relevance is the Cε3 domain, since it is the region through which IgE will bind to its cell surface receptor and is therefore the domain to be pharmacologically blocked. The Cε3 domain is the fraction of the IgE molecule that binds to membrane receptors, and it is the domain to which omalizumab (the anti-IgE antibody) will bind. The key epitope is therefore the Cε3 domain of human IgE [2,3].

## 3. Types of IgE

**Figure 2:**
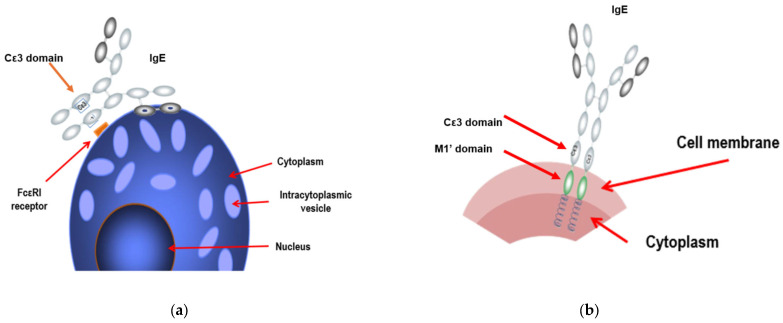
Types of IgE. There are two types: those secreted into the medium that bind to the cell receptor through the Cε3 domain (**a**) and the non-secreted (or membrane) IgE molecules, which are synthesized and expressed by the cell at the level of its cell membrane (**b**).

There are two types of IgE: the molecules released into the environment, and those expressed by the cell. In the sensitization process, the cell produces IgE molecules which are released and can be measured in a blood serum test, and these are the ones that will bind to their high- (FcεRI,) and low-affinity (FcεRII) receptors. There is, however, a second type of IgE: the molecules that are not released into the environment and which therefore are not measurable (that is, we cannot determine their presence in a blood test). These are the IgE molecules expressed by the cell. After the first exposure to the allergen and the initiation of the sensitization process, the individual’s B cells will now express IgE on their surface rather than IgG (these IgE molecules being very similar to the ones that they will produce and release). Unlike the IgE released into the environment, the B cells produce IgE that have an M1′ domain that allows them to anchor to the cell membrane, but not by binding to the FcεRI receptor. At this time, the B cells are already clearly differentiated (Figure 2a,b).

One might think that these cell-expressed IgE molecules do not play any role in the allergic process. However, this is not the case. Allergens that continue to penetrate the organism can follow three pathways: keeping the allergic cascade active by stimulating the process from the dendritic cells; favouring mast cell degranulation as each allergen molecule is captured by two IgE molecules (a cross-linking phenomenon already described above); or stimulating the Th2 pathway by interacting with these membrane IgE molecules. From the therapeutic point of view, although the basic aspect is omalizumab’s blockade of the Cε3 domain of the IgE secreted into the medium before binding to receptors, the drug can also bind to the Cε3 domain of the IgE expressed by B cells since, as we have said, they are anchored to the cell surface by the M1′ domain, and therefore the Cε3 domain is free. By binding to this membrane IgE, omalizumab provokes apoptosis of these cells [3].

## 4. Pathogenesis of the Allergic Reaction

When an individual is first exposed to an antigen, dendritic cells (macrophages located in the epithelium of the body) internalize it, process it, and present it to a T-lymphocyte through the major histocompatibility complex type II. In this process, the naïve T lymphocyte can become a T-helper 2 (Th2) or a T-helper 1 (Th1) lymphocyte. The relative amount of each type depends on both the antigen and the host (Figure 1) [2].

**Figure 3:**
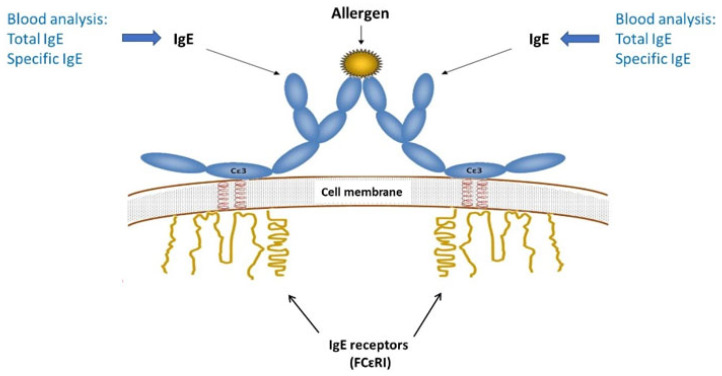
Acute allergic reaction: cross-linking phenomenon (taken from [5]). The individual is sensitized, produces IgE against the allergen to which s/he is sensitized, and these IgE molecules bind to their membrane receptors on mast cells and basophils. When a new allergen appears, two IgE molecules block the allergen, leading to changes in the intracellular component of the IgE receptor. This will cause mediator-loaded vesicles (in this case histamine) to be released into the environment.

When the antigen is an allergen, the lymphocyte differentiates into a Th2 cell capable of producing IL-4, which in turn promotes IgE synthesis by B cells. In addition to regulating IgE, IL-4 promotes IL-13 production in mast cells. In their membranes, mast cells contain the high-affinity receptor for IgE (FcεRI). When IgE binds to this receptor, it is ready to block the allergen to which it is sensitized. Up to this point (the sensitization phase), no clinical symptoms have appeared. When two or more IgE molecules that have bound to their receptor recognize the same allergen, they cause the cross-linking effect in the receptors, a phenomenon that triggers a series of biochemical signalling reactions culminating in mast cell degranulation [2,3,4] (Figure 3).

In the acute allergic reaction phase, the immediate mediators are released. This phase lasts approximately one hour. Subsequently, a second phase may occur after 4–8 h of exposure to the allergen. The chemotactic factors, IL-5, IL-3, IL-13, and cell growth factors released in the immediate inflammation increase eosinophil recruitment. Eosinophils release IL-5, which in turn perpetuates inflammation (the late reaction phase). This can lead to chronic inflammation in cases of continued exposure to the allergen. Occasionally, if the amount of IgE, eosinophils, and other mediators is very high, this process can persist without the need for an allergenic stimulus (chronic phase) [3].

## 5. Importance of Immunoglobulin E

In addition to the role described above, IgE collaborates in other aspects of the allergic reaction that are key elements in the pathophysiology of asthma. As we noted above, IgE binds to its high-affinity receptors, FcεRI receptors, on the surfaces of mast cells and basophils, through the Cε3 domain of its Fc fragment. The correlation between FcεRI expression on basophils and serum IgE levels is well established [6]. IgE itself [7] appears to up-regulate FcεRI expression in human basophils, probably by interacting with FcεRI. Recent data have suggested that IgE has some additional immunobiological effects. This molecule may promote mast cell survival through autocrine production of IL-6 [8]. IgE binds to dendritic cells and enhances allergen uptake and presentation to T cells [9]. Dendritic cells from patients with mild atopic asthma have been reported to bind significantly more IgE than cells taken from healthy individuals [10], and FcεRI receptors are known to be up-regulated on dendritic cells (as well as on eosinophils, mast cells, and macrophages) in patients with seasonal allergic rhinitis [11].

## 6. Omalizumab

The most important step in the production of a monoclonal antibody is the selection of the target. In the case of anti-IgE therapy, the goal is to obtain a specific monoclonal antibody against a key epitope of the IgE molecule. Omalizumab, known in early trials as rhu Mab-E25, is a humanized murine monoclonal antibody that recognizes the Cε3 domain of human IgE, the part of the molecule that binds to mast cell and basophil receptors. Once bound to these cell receptors, IgE undergoes a spatial transformation that favours allergen recognition. This spatial transformation also affects the Cε3 domain and renders it unrecognizable to omalizumab. As a result, omalizumab binds to free IgE but not to IgE bound to cellular receptors [12].

### 6.1. Pharmacological Effects of Omalizumab

**Figure 4:**
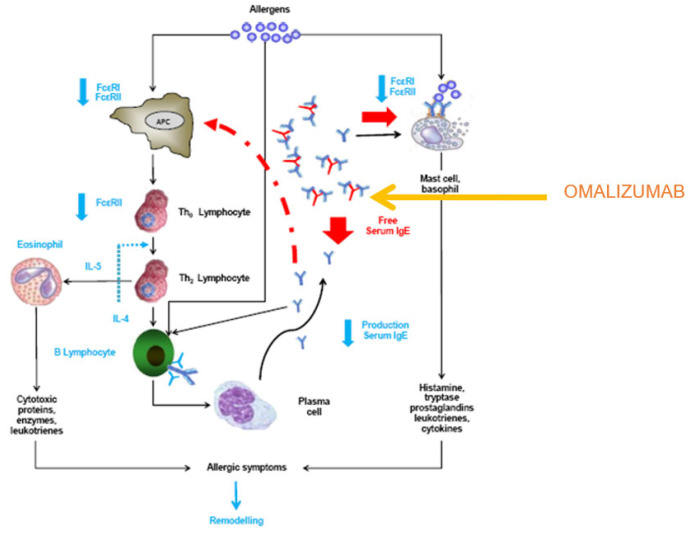
Effects of omalizumab. The steps of the allergic process are shown in black,. The steps blocked by the direct immunoglobulin E (IgE) blocking effect of omalizumab are shown in red. Blue indicates indirect immunomodulation mediated by the action of omalizumab, which causes the down-regulation of the cellular expression of FcεRI, and of FcεRII at different levels, the secretion of interleukin IL-4 and IL-5, and lowers eosinophil and B-lymphocyte levels, as well as IgE production. APC: antigen-presenting cell (modifed from [3], Domingo, C. *Drugs* **2014**, *74*, 521–533).

Initially, it was observed that omalizumab administration produces a rapid and substantial reduction in free serum IgE, which decreases by 99% within 2 h of administration. It also induces the down-regulation of FcεRI in basophils, dendritic cells, and monocytes within 7 days. At 3 months, the amount of FcεRI receptors on basophils decreases by up to 93% [13,14]. Therefore, the effect of omalizumab is to reduce both the amount of free serum IgE and the expression of FcεRI on mast cells and basophils. In addition, the decreased expression of FcεRI on dendritic cells may reduce allergen processing and presentation [15], and, consequently, may lead to reduced lymphocyte activation and decreased cytokine production from Th2 lymphocytes. Finally, omalizumab decreases serum, tissue, and sputum eosinophilia. This whole process is summarized in Figure 4.

Subsequently, it was reported that in addition to this direct effect, omalizumab has an immunomodulatory effect, which is shown below:

- Evident reduction of FcεRI expression in mast cells [16].
- Evident reduction of FcεRI expression in basophils [17].
- Decrease in histamine release by basophils [18].
- Reduction in basophils’ FcεRI-mediated capacity to release Th2 cytokines [19].
- Decrease in the number of dendritic cells (statistically significant in the case of myeloid dendritic cells (mDCs) and numerical in the case of plasmacytoid dendritic cells (pDCs)) [20].
- Decrease in the number of high-affinity IgE receptors in both pDCs and mDCs in patients with cat allergies [21].
- Decreased dendritic cell-dependent T cell proliferation in co-cultures stimulated with cat allergens [21].
- Significant decrease in the ability of mononuclear cells in culture to release IL-5 [22].

### 6.2. Clinical Development

Despite being the first biologic drug introduced, the development of omalizumab was very well programmed. In addition to its pharmacokinetic effects, the study of its pharmacodynamic effects was very thorough. The pivotal studies and the most important of the many real-life studies are described below.

#### 6.2.1. Studies Leading to the Development of Omalizumab

##### Pivotal Studies

Table 1 summarizes the most relevant data from pivotal studies focusing on patients with moderate/severe asthma. They show a notable and always significant reduction in exacerbations compared to placebo, and also demonstrate the drug’s safety. One of them also reports a marked reduction in inhaled corticosteroid use.

Undoubtedly, the most significant study is the INNOVATE study, which reported the following:

- A 50% reduction in severe exacerbations.
- A 44% reduction in emergency room visits.
- An improvement in the quality of life assessed by the AQLQ (asthma quality of life questionnaire), both in the overall score and in the various dimensions. The overall difference was statistically significant compared to placebo, although it did not reach the clinically relevant value of 0.5.

##### Real-Life Studies

Two real-life studies of omalizumab, one by Braunstahl (eXpeRience) [30] and one by Korn [31], stand out. The eXpeRience study was an international, open-label, single treatment arm, 2-year study involving 14 countries in Europe, the US, and Asia, and designed to evaluate the effectiveness of omalizumab. It included 943 patients with uncontrolled allergic asthma. Among the most noteworthy results were the following:

- The limitation in activities of daily living (ADLs) was reduced from 4.4 days per week to 1.3 and 1.2 at one and two years of follow-up, respectively.
- A decrease in the use of rescue medication that ran parallel to the improvement in ADLs. Consumption fell from 4.8 days per week to 1.8 and 1.6 at one and two years of treatment, respectively.
- The average annual days off work fell from 26.4 before the start of treatment with omalizumab to 3.5 and 1.0 days after one and two years of treatment with omalizumab, respectively.
- An increase in the percentage of patients without clinically relevant exacerbations (either severe or non-severe).

#### 6.2.2. Specific Aspects of the Development of Omalizumab

##### Fall in Exacerbations

The INNOVATE study [29] showed that the rate of clinically significant exacerbations (the primary efficacy endpoint), adjusted for an observed imbalance in exacerbation history, was 0.68 with omalizumab and 0.91 with placebo, representing a 26% reduction during the 28-week treatment phase. Without adjustment, a similar magnitude of effect (a 19% reduction) was observed, although it did not reach statistical significance. The CHOC study (Clinical and Histological impact of treatment with Omalizumab in severe allergic oral Corticosteroid dependent allergic asthma patients) [32] reported a 54% reduction in total exacerbations. For severe exacerbations, the INNOVATE study [29] showed an annualized reduction of 50%. In the CHOC study [32], none of the patients treated with omalizumab had severe exacerbations during follow-up, and exacerbation duration was reduced by half.

Two real-life studies involving oral corticosteroid (OC)-dependent patients (the eXpeRience registry [30], with 28.1% of patients receiving OC, and the Xpertise trial [31], with 46%) showed reductions in exacerbations of more than 80%.

In addition to exacerbations, some studies have measured emergency department visits or hospitalizations. The INNOVATE study [29], in which OC-dependent patients represented 22% of the sample, showed a 44% annual reduction in emergency room visits. In a real-life study, Molimard et al. [33] found a 65% reduction in annual ED visits after treatment with omalizumab. In Germany, in a population of 280 patients, 46% of whom were cortico-dependent, Korn et al. [31] reported an 82% reduction in exacerbations and a 78% reduction in hospitalizations.

##### Corticosteroid Sparing Capacity

The Cochrane review [34] concluded that treatment with omalizumab was associated with a significant possibility of reducing inhaled corticosteroid doses or of withdrawing them completely. The mean dose reduction was −118 μg beclomethasone dipropionate equivalents. In the subgroup of patients receiving OCs, it was unclear whether this benefit occurred. In a subgroup analysis in an open-label parallel-group study, Siergiejko et al. [35] found that at week 32, 62.7% of patients in the treated group were able to discontinue or reduce OCs compared with 30.4% of controls. The real-life eXpeRience study [30] found a relative reduction of 50% at month 24, and the APEX study, a retrospective study conducted in the UK, found a 34% reduction in the mean total amount of prescribed OCs per year and complete cessation of OC use after 12 months in 48% of patients [36]. In a 2-year prospective observational study, Domingo et al. [37] reported that omalizumab allowed OC withdrawal in 74.2% of patients. Real-life studies are usually understood to obtain better results than randomized clinical trials, but the CHOC study [32] presented results of the same order as the best real-life studies. In this open randomized study, it was observed that 12 patients (75%) in the omalizumab group and only one (7.7%) in the control group were able to cease OC treatment.

##### Biomarkers Predicting Response to Omalizumab

The biomarkers that have been evaluated are the exhaled fraction of nitric oxide (FeNO), peripheral blood eosinophils, peripheral blood immunoglobulin E (IgE) concentration, and, in experimental studies, the blood periostin value. An early study by Djukanovic [14] in 45 patients with mild/moderate asthma, who were divided into two groups of 22 (omalizumab) and 23 (placebo), showed at 16 weeks that treatment with omalizumab decreased the number of eosinophils in patient sputum (from 6.6% to 1.7% in the treated group; *p* = 0.05) versus a non-significant decrease in the placebo group (from 8.5% to 7.0%). This decrease was also observed in bronchial biopsy specimens.

The EXTRA study [38] found the patients with the lowest incidence of exacerbations to be those with FeNO values ≥ 19.5 ppb, eosinophils ≥ 260 µL, and periostin ≥ 50 ng/mL.

Eosinophil rates may be elevated in allergic patients without the patient exhibiting an eoosinophilic phenotype. There is some controversy as to whether this biomarker can condition the response to treatment with omalizumab. A study by Casale et al. [39] also showed that the baseline eosinophil level, as well as other clinical markers, predicted response to omalizumab. Patients with a history of frequent ED visits, hospitalizations, FEV1 (%) values < 65%, beclomethasone dipropionate doses of ≥600 µg, and who use long-acting beta-agonists (LABAs) are the best responders to omalizumab. The STELLAIR retrospective study [40], conducted in France, included 872 patients (723 adults and 149 non-adults aged 6–17 years) with severe allergic asthma who were treated with omalizumab by 78 physicians. The mean eosinophil count was ≥300 cells-µL-1 in 52.1% of the adults and in 73.8% of the children/adolescents. The percentage of responders did not vary in the subgroups established according to the baseline eosinophil value, leading the authors to suggest that the eosinophil level is not a good predictor of response to omalizumab.

Finally, it has been speculated that omalizumab may have a long-term effect on IgE synthesis. Lowe and Renard [41] obtained data on omalizumab and free and total IgE from an epidemiological study and from six randomized, double-blind, placebo-controlled trials in patients with allergic asthma. IgE production and clearance were modelled, and it was concluded that after five years of treatment, IgE production decreases. In a study in which free IgE and total IgE (free + blocked with omalizumab) were measured, Domingo et al. [42] observed a decrease in IgE production. This result appeared to justify the discontinuation of treatment after five years, but this biological observation did not correspond to the clinical findings, and cannot therefore be used as a criterion for stopping treatment.

### 6.3. Long-Term Tolerance

When a new drug is introduced, one of the key aspects is its safety. The EXCEL study [43,44], conducted at the request of the FDA, was a post-marketing study that included 4972 patients treated with omalizumab and 2867 patients who were not treated. It demonstrated the safety of the drug, but raised doubts about the incidence of neoplasms, which appeared to be higher in the treated group. The increased risk was attributed to the fact that the treated group was followed for a longer time. Subsequently, a study by Long et al. [45] showed that omalizumab treatment does not increase the incidence of malignancies. A recent retrospective study in 45 patients with a mean follow-up of 10.6 ± 1.2 years [46] has also shown the effectiveness and safety of the drug.

### 6.4. Withdrawal of Treatment

An early study showed that after omalizumab withdrawal, symptoms reappear and are accompanied by an increase in free IgE [47]. Subsequently, Molimard et al. [48] observed a loss of asthma control in 55.7% of patients 13 months after treatment withdrawal. After six years of treatment, Kupryś-Lipińska and Kuna [49] observed that 9/11 (81.8%) presented exacerbations within five months of treatment withdrawal. A study by Nopp et al. [50] in a population of cat epithelium allergic individuals demonstrated that three years after withdrawal from a six-year treatment programme with omalizumab, there was no loss of respiratory function. In the XPORT study [51], a randomized study with two treatment groups (a minimum of five years with omalizumab versus placebo) showed that patients who continued with omalizumab had better asthma control during the 52 weeks of the study. Vennera et al. [52] observed that despite five years of treatment with omalizumab, the number of patients with severe exacerbations increased over time, with 39% of patients presenting severe exacerbations four years after discontinuation of treatment. The OMADORE study [53] is the only study that has proved able to identify patients in whom omalizumab can be safely withdrawn. Among the patients who met the clinical stability criteria and whose FEV1% did not deteriorate after 18–24 months of treatment, those who tolerated the decrease did so between 18–40 months after starting treatment; they tended to be younger and have better GETE, improved FEV1% after the first dose of omalizumab, reduced oral corticosteroid use, and FeNO values < 50 ppb. With this protocol, omalizumab could be withdrawn in approximately one third of patients, without severe exacerbations occurring during the following 30 months. More recently, in a retrospective study in France based on information from a national health service data registry, 16,750 adults and 2452 children (age ≥ 6 years) were identified with median periods of omalizumab treatment before withdrawal of 51.2 and 53.7 months, respectively. Among the adults who discontinued omalizumab when their asthma was controlled, 70%, 39%, and 24% remained controlled and did not resume omalizumab treatment one, two, and three years after discontinuation, respectively [54].

### 6.5. Particular Effects of Omalizumab

In addition to the descriptions above, numerous studies have revealed other unexpected effects of the drug.

#### 6.5.1. Omalizumab and Remodelling

Information on remodelling and biologic treatments in patients with severe asthma is limited and often indirect. Omalizumab has been shown to decrease endothelin-1 clearance in exhaled air [55], reduce bronchial wall thickness, and increase the bronchial endoluminal area [56]. Two ex vivo studies have shown that the presence of IgE induces extracellular matrix and collagen deposition and antagonizing IgE reverses these effects [57,58]. The only study to demonstrate the reversibility of remodelling in the ciliated epithelium (including the reappearance of cilia) and reductions in intracellular spaces and in the thickness of the basement membrane is the CHOC study [32].

#### 6.5.2. Omalizumab and Infections

Among the factors that predispose patients to the triggering of asthma exacerbations are respiratory infections of viral origin, which lead to peaks of exacerbations throughout the year. This is clearly evidenced in the study by Johnston et al. [59].

**Figure 5:**
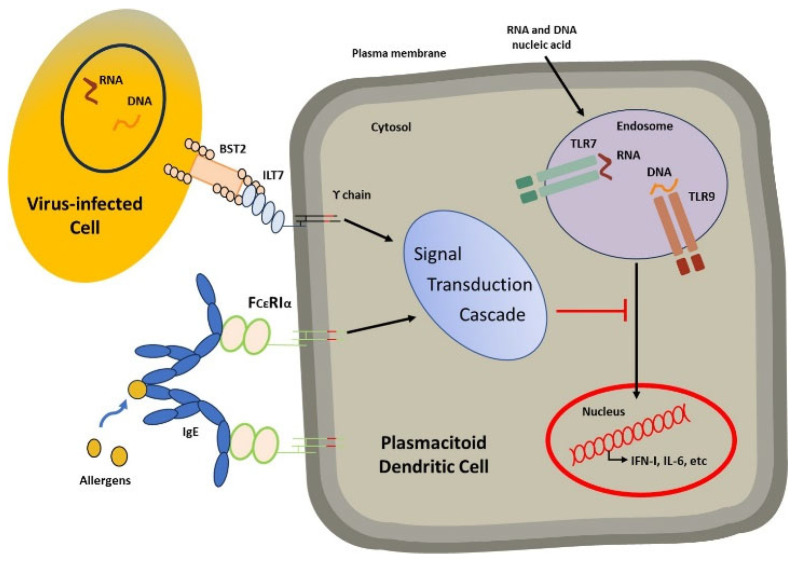
Modified from Lynch et al. [61]. The figure shows how IgE molecules bind to their receptors and are ready for the cross-linking phenomenon to occur in the presence of allergens. This will activate the cascade that blocks the command sent from the toll-like receptors for the nucleus to synthesize interferon.

The possibility that there might be a relationship between the presence of IgE molecules and their binding to their high-affinity receptors and lung infections was raised when a decrease in interferon production was observed in patients with asthma infected by the influenza virus; in this situation, there was an inverse relationship between IgE blood concentration and interferon production [60]. It was suggested that the production of interferon by plasmacytoid dendritic cells might be down-regulated due to the binding of free IgE molecules to their high-affinity receptors upon the activation of a cascade that blocks the order sent from toll-like receptors stimulated by the presence of viral genetic material (Figure 5).

If all this is true, it makes sense to examine whether blocking IgE might stop the cross-linking phenomenon, and whether interferon production in the presence of viral genetic material would therefore remain intact. This possibility was clinically confirmed by the ICATA study [62], which showed that treatment with omalizumab improves the control of allergic asthma, prevents seasonal exacerbations, and reduces the consumption of inhaled corticosteroids.

The PROSE study confirmed that the addition of omalizumab 4–6 weeks before the start of the school year and maintained for the following four months reduced exacerbations more than in the placebo group and in the group that received a reinforcement of inhaled corticosteroids, since it contributed to restoring the defensive mechanisms [63]. This benefit also seems to extend to asthmatic children with more severe disease [64].

### 6.6. Omalizumab and Pregnancy

Information on the use of omalizumab during pregnancy is limited. However, the Expect registry [65] collected information from 230 pregnant women (64.9% with severe asthma and 35.1% with moderate asthma) treated with omalizumab from 2006 to 2017 in the US and compared these data with those obtained from a cohort of 1153 women from Quebec (21.2% with severe asthma and 78.8% with moderate asthma) who were not treated with omalizumab, which served as a control. The study concluded that omalizumab can be safely administered to pregnant women, and that it does not increase the risk of malformations in the foetus or the risk of small size for gestational age.

### 6.7. Indication of Omalizumab

The drug is indicated in patients with severe allergic asthma. Certain factors regarding the initial indication and subsequent modifications should be borne in mind (see Table 2) [3].

It has also been observed that omalizumab is effective in patients with severe allergic asthma who have been sensitized to seasonal allergens [66]; however, this indication does not appear on the technical data sheet. Other exploratory indications include the administration of the drug as a preventive treatment in cases of immunotherapy for venoms [67] or in exercise-induced anaphylaxis [68]. These prescriptions should be made in specialized units.

## 7. Conclusions

Omalizumab has had an exemplary theoretical and practical development. By blocking IgE, a molecule that is undoubtedly central to the development of allergic reactions, it has led to a better understanding of the Th2 pathway and has contributed to the concept of T2 inflammation. The exemplary development of omalizumab over the past 20 years has enabled shorter development times for other monoclonal antibodies that block molecules such as the interleukins IL5, IL4, and IL13 and the alarmins TSLP, IL33, and IL25.

## Figures and Tables

**Table 1:** Summary of pivotal studies.

Author	Year	Study	Asthma Severity	Nº of Patients	Efficacy Variable	Results	Duration (Weeks)
Busse [23]	2001	008	Severe	525	Exacerbations	↓ 48%	28
Solèr [24]	2001	009	Moderate–Severe	546	Exacerbations	↓ 58–52%	52
Holgate [25]	2004	011	Severe	246	ICS saving	74% of patients ↓ FTC ≥ 50%	32Yes, this
Vignola [26]	2004	SOLAR	Moderate–Severe	405	Exacerbations AQLQ	↓ 38%	28
Ayres [27]	2004	ETOPA	Moderate–Severe	312	Exacerbations/ worsening of asthma	↓ 61%	52
Bousquet [28]	2004	ALTO	Moderate–Severe	1899	Safety		24
Humbert [29]	2005	INNOVATE	Severe	419	Exacerbations	↓ 50%	28

ICS: inhaled corticosteroids. FTC: fluticasone.

**Table 2:** Criteria for indication of omalizumab at the start of marketing and subsequent modifications.

	At Marketing	Post-Marketing Modification
Age (years)	≥12	≥6
Allergy sensitization	Positive skin prick test or in vitro reactivity to at least one perennial aeroallergen	Positive skin prick test or in vitro reactivity to at least one perennial aeroallergen
Baseline immunoglobulin E level	≥30–700 kU/L	≥30–1500 kU/L
Monthly calculated omalizumab dose	≤750 mg	≤1200 mg
Asthma severity	Severe or inadequately controlled asthma	Severe or inadequately controlled asthma
Re-evaluation	After 16 weeks of treatment	After 16 weeks of treatment
Long-term treatment withdrawn	Not specified	Not specified
